# Depth-Aware Networks for Multi-Organ Lesion Detection in Chest CT Scans

**DOI:** 10.3390/bioengineering11100998

**Published:** 2024-10-03

**Authors:** Han Zhang, Albert C. S. Chung

**Affiliations:** Department of Computer Science and Engineering, The Hong Kong University of Science and Technology, Clear Water Bay, Hong Kong, China

**Keywords:** computer-aided diagnosis, convolutional neural network, multi-organ lesion detection

## Abstract

Computer tomography (CT) scans’ capabilities in detecting lesions have been increasing remarkably in the past decades. In this paper, we propose a multi-organ lesion detection (MOLD) approach to better address real-life chest-related clinical needs. MOLD is a challenging task, especially within a large, high resolution image volume, due to various types of background information interference and large differences in lesion sizes. Furthermore, the appearance similarity between lesions and other normal tissues demands more discriminative features. In order to overcome these challenges, we introduce depth-aware (DA) and skipped-layer hierarchical training (SHT) mechanisms with the novel Dense 3D context enhanced (Dense 3DCE) lesion detection model. The novel Dense 3DCE framework considers the shallow, medium, and deep-level features together comprehensively. In addition, equipped with our SHT scheme, the backpropagation process can now be supervised under precise control, while the DA scheme can effectively incorporate depth domain knowledge into the scheme. Extensive experiments have been carried out on a publicly available, widely used DeepLesion dataset, and the results prove the effectiveness of our DA-SHT Dense 3DCE network in the MOLD task.

## 1. Introduction

Computer-aided diagnosis (CADx) plays a crucial role in medical image analysis. In recent years, convolutional neural network (ConvNets) based deep learning methods have demonstrated superior detection accuracy over traditional statistical learning approaches, which rely solely on handcrafted features. A lesion detection system is an essential part of CADx. Many research works have focused on enhancing lesion detection tasks [1,2,3,4,5,6,7,8,9,10,11,12]. Most research works have used ConvNets-based methods and achieved satisfying detection sensitivity and precision within a limited scope of detection.

The majority of current lesion detection CADx systems [1,2,3,4,5,6,7,8,9,10,11,12] are designed to detect one or two specific diseases within a particular organ. Although these disease-specific CADx systems can be integrated into modular solutions as an “ensemble of experts”, the significance of front-line screening universal lesion detection CADx systems should not be overlooked. Therefore, the universal lesion detection (ULD) task [1,2,6,13] has been proposed. The ULD task aims to detect nearly all kinds of lesions from medical images to deliver more clinical value. This is especially true when diagnosing CT scan slices with multiple organs and different kinds of tissue because it allows clinicians and medical researchers to identify multiple types of detected lesions.

To further improve the ULD CADx system, in this paper, we aim to detect and specify lesion types in chest CT scans by introducing the multi-organ lesion detection (MOLD) framework. Unlike a ULD task, which only distinguishes between lesions and non-lesions, our MOLD task aims to detect and specify different kinds of lesions, which can be located in different organs. The most obvious difference between ULD and MOLD tasks is that the ULD task is a binary class detection task, while the MOLD task is a multiple class detection task. MOLD is a crucial but underdeveloped problem in CADx. Our approach, by contrast, is designed to detect multiple types of lesions across different organs within the same scan, thereby aligning more closely with the holistic diagnostic process used by clinicians. One reason is that diseases may have complications. For example, hepatorenal syndrome (HRS) is a common complication of advanced cirrhosis, characterized by renal failure and major disturbances in circulatory function [14]. The simultaneous interpretation of the liver and kidney in medical images can greatly enhance the detection of this serious complication and possibly help deliver effective treatments to patients. Another reason is that some metastatic carcinoma is prone to metastasis and spread. For example, the most frequent cause of cancer death is lung cancer [15]. At the time of the initial diagnosis, about 50% of all lung cancer patients have distant metastasis [16]. In patients with lung cancer, the bone, brain, liver, and adrenal glands can be the most common sites of metastatic diseases [17]. Detection of complications and spread of metastatic carcinoma to other organs can significantly help experts diagnose and determine different stages of the disease. For those cancer patients with metastases, clinicians will simultaneously identify the locations of both primary and metastatic lesions during the diagnosis process. Then, clinicians can combine the morphology and characteristics of those two types of lesions to provide more accurate and comprehensive cancer staging and diagnoses. For instance, if lung cancer has spread to lymph nodes, clinicians first determine the position of the lesions in the lymph nodes. If the location is close to the primary lesion, it suggests that the tumor is still partial, in the early stage or mid-stage, and surgery may be used for removal. If distant lymph node metastasis occurs, it may be an advanced tumor, and a lifetime treatment regimen should be systemically considered rather than surgery. The variety in complications and metastatic carcinoma makes the elaborated diagnosis time-consuming for clinicians. Therefore, the MOLD system will become very useful in practice. In addition, our proposed MOLD system can also help clinicians in patient shunting, disease screening, and other tasks.

There has been some research on the ULD task, especially with the DeepLesion dataset, a large dataset with 32,120 chest CT scans that include eight different types of lesions, including bone, abdomen, mediastinum, liver, lung, kidney, soft tissue, and pelvis [18]. Based on this dataset, Yan et al. [1] proposed a 3D context enhanced (3DCE) region-based CNN lesion detection model, which can combine the advantages of both 2D and 3D networks through concatenating the 3D features on the feature map level. Based on this 3DCE framework, Tao et al. [2] proposed a dual-attention mechanism, Li et al. [4] proposed a multi-view feature pyramid network with a position-aware attention network, and Zhang et al. [3] introduced a dense auxiliary loss mechanism embedded in a dense feature extraction network. However, each of these works has the following limitations. Therefore, we aim to tackle these challenges. First, all methods mentioned above focus on the ULD task. The lack of output categorical information will make diagnosis difficult. Compared to the ULD task, the detection results obtained from the MOLD task can provide categorical information, which the original ULD task does not provide. Second, the backpropagation in the training process is not optimized, which makes the over-fitting obvious. This is particularly true for the MOLD task, as only around 30% of the original DeepLesion dataset samples have a multi-organ category label that can be employed for the MOLD task. Third, these three methods do not consider the depth information of the CT slices, which can reliably indicate which organs are included in the CT slices [19]. The depth scores predicted from the depth score regressor can help improve detection performance. The depth score regressor establishes a coordinate framework for the human body and generates a continuous score for each axial slice, indicating the normalized position of the body part within the slice. For example, lung lesions and kidney lesions cannot exist in the same transverse CT slice owing to their different position of the body part in the slice. As a result, there will be significant differences in their depth scores. Therefore, classification errors caused by similar texture features can be avoided by finding inconsistencies between lesion types and depth information. This can improve the accuracy of detection as well as strengthen the robustness of the lesion detection system, especially in the MOLD task. More details of the depth score regressor can be found in Section 2.6.

With MOLD, the above problems will be addressed and tackled. This paper further optimizes the backpropagation process in our proposed framework. Based on the 3DCE framework [3], we improve the backpropagation of the training process and incorporate depth information in the proposed network. First, we propose a skipped-layer hierarchical training (SHT) mechanism to optimize the dense auxiliary losses (DAL) [3]. Utilizing different losses in different training stages can produce a positive trend in the backpropagation process. Second, we introduce a depth-aware (DA) mechanism that considers the CT depth coordinates so as to extract information from the CT slices in three dimensions. Our DA-SHT 3DCE model is a two-stage convolutional neural network (CNN). It utilizes the VGG-16 model [20] as the feature extractor and uses a 3DCE region-based fully convolutional network (R-FCN) [21] as the backbone. To evaluate the effectiveness of our model, we have performed extensive experiments on a publicly available, widely-used public dataset, i.e., DeepLesion [22]. Section 4 presents the results, demonstrating our proposed model’s effectiveness. To summarize, the contributions of our work are as follows:

- A groundbreaking attempt to perform the MOLD task on the DeepLesion dataset, which not only distinguishes lesions from non-lesions but also detects and specifies eight different kinds of lesions so as to greatly enhance the clinical value.
- Improving the network architecture in [3] by adapting DALs through the SHT mechanism to increase the robustness of the backpropagation process and reduce over-fitting.
- Taking domain knowledge into consideration and adding the DA mechanism to extract features in three dimensions benefits the lesion type classification process at a minimal cost.

In this paper, we aim to propose a multi-organ lesion detector to better address real-life chest-related clinical needs. Specifically, we try to tackle the following challenges: First, the practicability of the current CADx system in clinical diagnosis is limited. Second, ignoring the depth scores of the CT slices will lose implicit information and decrease the robustness of the MOLD system. Third, the training process through the existing dense auxiliary loss (DAL) mechanism of the loss function is sub-optimal. In conclusion, a novel multi-organ lesion detector is proposed. Extensive experiments have been carried out on a publicly available, widely used DeepLesion dataset, and the results prove the effectiveness of our DA-SHT Dense 3DCE network in the MOLD task.

## 2. Related Work

### 2.1. Object Detection

Object detection is a widely studied topic in the computer vision field. Some classic frameworks for object detection are also exploited in CADx lesion detection, such as R-CNN [23], Fast R-CNN [24], Faster R-CNN [25], R-FCN [21], and so on. From R-CNN to Fast R-CNN to Faster R-CNN, the efficiency and effectiveness are continually improving. The largest difference between an R-CNN and a Fast R-CNN is that a Fast R-CNN processes the four steps required for object detection: candidate region generation, feature extraction, category classification, and bounding box regression in one unified neural network. It also runs the model on a GPU, which greatly improves the efficiency of the operation [26]. A Faster R-CNN network is built based on the Fast R-CNN network design, but it improves the process of candidate region generation by introducing a region proposal network (RPN), which is a module for generating proposals. An R-FCN network is a transformation based on the framework of Faster R-CNN. The region of interest (ROI) pooling layer is replaced by the position-sensitive region of interest (PSROI) pooling layer. Since the ROI pooling layer does not contain location information, the location information is added before pooling to specify different score maps that are responsible for detecting different locations of detected objects. After pooling, the score maps obtained from different locations can be combined to reproduce the original location information. In order to prove the effectiveness of our proposed method, we use the Faster R-CNN and R-FCN models as the baseline methods for comparison.

In recent research, transformers have shown exceptional utility in object detection tasks. Recent studies have showcased transformative applications of transformers in object detection. For instance, Song et al. [27] developed a Vision and Detection Transformer (ViDT) that optimizes accuracy and latency trade-offs on the Microsoft COCO benchmark dataset. Additionally, Liang et al. [28] introduced DetectFormer, which improves detection performance in traffic scenes by leveraging global information and category-assisted decoders.

### 2.2. FCN-Based Models

Our Novel Dense 3DCE R-FCN model is proposed based on the R-FCN model. The R-FCN [21] model evolved from the Fully Convolutional Network (FCN) [29] to address the limitations in object detection tasks. FCN is designed for pixel-wise classification, making it efficient for segmentation tasks but less suitable for object detection due to its lack of region-specific attention. R-FCN introduces a region-based approach by incorporating Region Proposal Networks (RPNs) and position-sensitive score maps. This architecture enables the model to generate region proposals and focus on specific object regions, improving detection accuracy and efficiency. R-FCN achieves a balance between the computational efficiency of FCN and the precision of region-based methods like Faster R-CNN.

FCN model has been used for segmentation tasks in many medical applications. For example, Zhou et al. [30] proposed the MDSU-Net model. The MDSU-Net model incorporates dual attention mechanisms and depthwise separable convolutions to enhance feature extraction and reduce model complexity for medical image segmentation. In addition, Karthik et al. proposed a novel method in [31]. The proposed method integrates an attention-gated mechanism with an FCN to enhance ischemic lesion segmentation from multimodal MRI. By incorporating attention gates into the FCN’s decoder, the model selectively filters and emphasizes relevant features, significantly improving segmentation accuracy compared to traditional FCN and other existing methods. The authors in [32] proposed the SAN-Net model. The SAN-Net model introduces a self-adaptive normalization mechanism to enhance stroke lesion segmentation from multimodal MRI. Unlike traditional FCN, SAN-Net employs Masked Adaptive Instance Normalization (MAIN) for dynamic standardization and a site classifier with a gradient reversal layer for site-invariant representation learning, significantly improving generalization to unseen sites.

The primary distinction between the aforementioned FCN-based model and our model lies in the target task. Our model is specifically designed for lesion detection in CT cases and does not provide segmentation results. Additionally, our model is based on the R-FCN architecture, meaning that network training is focused on region proposals rather than the entire image.

### 2.3. Lesion Detection in CADx

The methods mentioned above are widely used in object detection in natural images and lesion detection in a CADx system. Although these methods are practical and useful, they need to be tailored for lesion detection tasks because medical images are different from natural images. For example, although images in the ImageNet dataset [33] and the DeepLesion dataset [22] are all in 2D, CT slices in the DeepLesion dataset are always in image volume format, which can give additional three-dimensional, spatial information for the detection task. Thus, these networks are inadequate for CT slices to consider 3D information. Therefore, we use the 3DCE network [1], which can consider 3D context information for detection performance enhancement. It integrates the crucial 3D context information as it includes the neighboring CT slices to the 2D detection network, and then it concatenates the feature maps together to perform final predictions on the categories and bounding boxes. This scheme allows the 3D context information to be better utilized, and the performance is much better than using a single CT slice alone. To preserve these advantages and prove the effectiveness of our method, we exploit the 3DCE network as the backbone of our method and significantly enhance it to perform both ULD and MOLD tasks. At the same time, we also used it as the baseline method and compared it with our new DA-SHT Dense 3DCE model.

Based on the 3DCE model, some existing methods for the lesion detection task exist. For example, the attention theme proves to be effective in a CADx system because it can better simulate the workflow of the clinicians. For instance, Tao et al. [2] proposed a method to add 3D contextual attention and a spatial attention theme to the 3DCE network mentioned above so as to ensure that attention exists not only between the slices but also inside them. These attention themes allow 3D contextual information to be used without retraining the large 3D ConvNets. Zhang and Chung [3] proposed a framework to address the shallow layer supervision and feature combination problems. We formulate our new method based on our former proposed method [3] with significant extensions and use the new model on both ULD and MOLD tasks. The extensions are mainly three-fold. First, our method adopts the SHT scheme to better utilize DALs in [3], which can reduce the parameters and complexity of the model as much as possible under the premise of maintaining model performance. Second, the new method considers the depth score of CT slices, which is an unsupervised process and does not need extra annotations. Third, we extend the adapted tasks from binary lesion detection to multi-organ lesion detection.

Besides using the 3DCE-based frameworks, several applications use other frameworks to work on the lesion detection task and use an attention scheme. For instance, Li et al. [4] proposed a framework that combines a multi-view feature pyramid network (FPN) with a channel-wise position-aware attention module. The multi-view features are obtained from slices rendered with various window widths and levels, while the position-aware attention is employed with a module similar to the Convolutional Block Attention Module (CBAM). The model presented in [5] also utilizes an attention scheme to build an improved RetinaNet framework for the ULD task. Tang et al. [6] proposed the Universal Lesion Detector (ULDor) model, which utilizes the Mask R-CNN framework in [34] as the backbone. This framework needs pixel-level annotations for training, so the pseudo masks are generated to fit that need. It also uses negative example mining to further improve performance. Zhang et al. [7] proposed a Modified Pseudo-3D Feature Pyramid Network (MP3D FPN) that leverages depthwise separable convolutional filters and a group transform module (GTM) to efficiently extract 3D context-enhanced 2D features for universal lesion detection in CT slices. Xu et al. [8] proposed a PAC-Net, which integrates a multi-pathway Feature Pyramid Network with position attention guided connections and vertex distance IoU, enhancing the accuracy and efficiency of 3D medical image detection by addressing position offset and IoU-based loss degradation.

The abovementioned methods mainly concentrate on universal lesion detection, which distinguishes lesions from non-lesions. Although various multi-class lesion detection methods have been proposed, for example, [9,10,11], all these methods only focus on one single organ, such as the heart, brain, and skin, which suffer from limitations in the scope of the available scenes of the model. In recent years, more researchers have pointed out the importance of multi-organ-based medical image analysis. For example, Xu et al. proposed a multiple-organ localization framework [12], which uses a 3D region proposal network. However, there are only very few related recent works in this direction. Compared with other single organ-based lesion detectors that detect the target organ in all slices to assemble the final bounding boxes with confidence scores alone, our new method is implemented in a multi-organ manner by taking full advantage of the spatial depth and category information in CT scans. As such, our method performs the MOLD task and can output both lesion type and confidence score simultaneously.

Besides these lesion detection models, there are also some methods that work on the DeepLesion dataset [22] and focus on other tasks. For example, the model presented in [18] uses a triplet network, which aims at content-based lesion retrieval and intra-patient lesion matching.

### 2.4. Multi-Level Feature Reuse and Supervision

Densenet [35] is a classic framework that has been widely used in recent years. Its main advantage is that it allows feature reuse, achieved through connecting features on channels. Inspired by Densenet, we realize the importance and effectiveness of feature reuse in a network, especially in the ULD and MOLD tasks. Due to the huge differences in lesion sizes, nearly all resolutions of the feature maps have their own utility. This means that shallow, intermediate, and deep information cannot be ignored. Therefore, in this paper, inspired by the Dense 3DCE R-FCN structure in [3], a CNN model training process is a coarse-to-fine model fitting process. We need to employ different supervision mechanisms in different training stages to better adapt to this process. Therefore, we propose a hierarchical feature reuse and supervision scheme.

### 2.5. Hidden Layer Supervision

Besides feature reuse, supervision in the training process is also crucial. As shown in [36], a deeply supervised network (DSN) introduces a “companion objective” to the individual hidden layers in addition to the overall objective at the output layer (a different strategy than the layer-wise pre-training). As a new variant of the DSN, Sun et al. [37] proposed a Deeply-supervised Knowledge Synergy (DKS) scheme to improve the generalization ability of the CNNs. The scheme introduces a novel synergy loss, focusing on the knowledge-matching problem among the supervision loss pairs. Inspired by these works, we introduce the SHT strategy and embed it in the Dense 3DCE R-FCN network. To prove the efficiency of the proposed method, the DSN and DKS methods mentioned above are reproduced as our baseline methods.

### 2.6. Depth Information Obtainment

Obtaining the depth scores of the CT slices is similar to a body part recognition task. The models for this task are mainly divided into two categories: supervised models and unsupervised models. Yan et al. [19] proposed an unsupervised body part regression model, which can output a normalized depth score for each CT slice. The training process of this regression model behaves like a self-organization process because these normalized depth scores are all learned from the inter-slice relationships. This means that no extra work is required to annotate the CT slice volumes. As such, we deploy this regression model to assist in lesion detection and enhance the detection performance, especially the classification process, by providing depth information at the lowest cost.

### 2.7. Research Objectives and Research Questions

Building on these foundations, our research aims to develop a novel multi-organ lesion detection (MOLD) approach that addresses these challenges and meets real-life clinical needs more effectively. The primary objectives of our study are to:

- Develop an advanced deep learning model that integrates depth-aware (DA) and skipped-layer hierarchical training (SHT) mechanisms to enhance lesion detection accuracy.
- Evaluate the performance of the proposed model on a large, publicly available dataset (DeepLesion) and compare it to existing models.

To achieve these objectives, our study focuses on several key aspects:

- The integration of depth-aware mechanisms significantly improves the detection accuracy of multi-organ lesions in chest CT scans.
- The implementation of skipped-layer hierarchical training has a substantial impact on the performance of the Dense 3D context-enhanced (Dense 3DCE) network in detecting lesions of various sizes and appearances.
- The proposed DA-SHT Dense 3DCE model demonstrates superior detection accuracy and computational efficiency compared to existing state-of-the-art models.

By addressing these aspects, we aim to contribute to the development of more effective and robust CADx systems for multi-organ lesion detection.

## 3. Methodology

**Figure 1:**
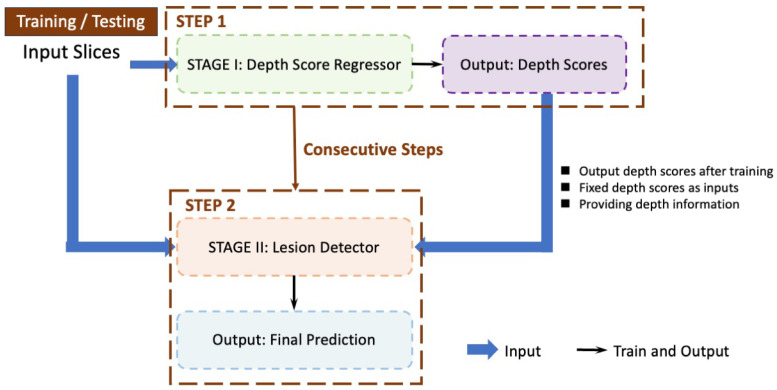
The pipeline of training and testing procedures. The regressor outputs are fixed and passed to the detector as inputs. The training of the regressor and the detector are consecutive steps. The regressor is trained first, and then the detector is trained.

We propose a two-stage lesion detection network. Figure 1 provides a detailed illustration of the sequential steps involved in our proposed detection approach. During the testing phase, the workflow mirrors that of the training phase. The process is divided into two main stages:

- Stage I: Depth Score Regressor (Step 1)—This initial stage, outlined by the green dashed box, takes the input slices and processes them to output depth scores. These depth scores are crucial for the next stage and are produced after training.
- Stage II: Lesion Detector (Step 2)—The second stage, marked by the orange dashed box, uses the depth scores generated from Stage I as fixed inputs to detect lesions. This stage outputs the final prediction of lesions within the CT slices. The process flows consecutively from Stage I to Stage II, as indicated by the brown arrows.

**Figure 2:**
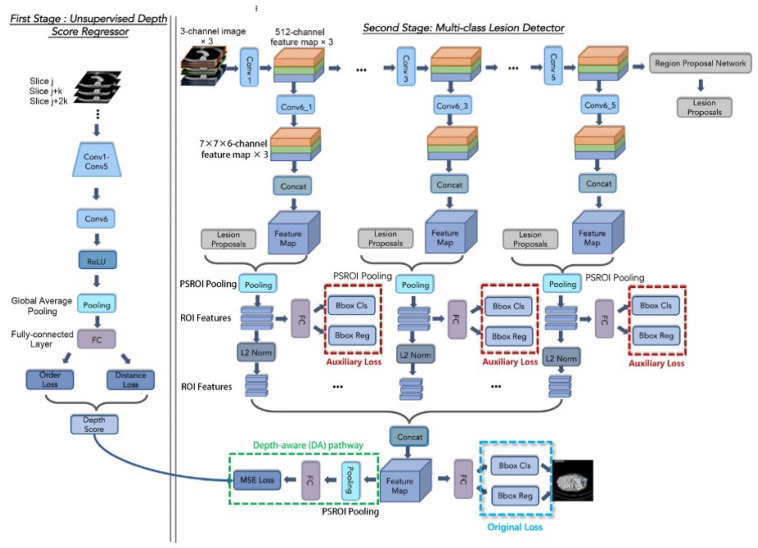
The architecture of our DA-SHT Dense 3DCE Network, using our Novel Dense 3DCE R-FCN as the backbone framework. The training of the regressor and the detector are consecutive steps. The regressor is trained first, and then the detector is trained.

Although the whole framework is called a two-stage framework, the detection network in the second stage is end-to-end training and is easy to deploy. The detailed architecture of the Depth Score Regressor presented in Stage I of Figure 1 further elaborated on the left side of Figure 2, while the right side of Figure 2 provides an in-depth breakdown of the Lesion Detector’s internal structure. In Figure 2, we provide a comprehensive breakdown of the Regressor’s internal structure, showcasing each individual neural network block and layer involved in processing the input slices to generate the depth scores. It also illustrates each individual neural network block and layer involved in processing the depth scores and input slices to generate the final lesion detection output. This detailed depiction aims to offer a clear understanding of the components and operations within the regressor and the detector, complementing the overall process flow illustrated in Figure 1.

**Figure 3:**
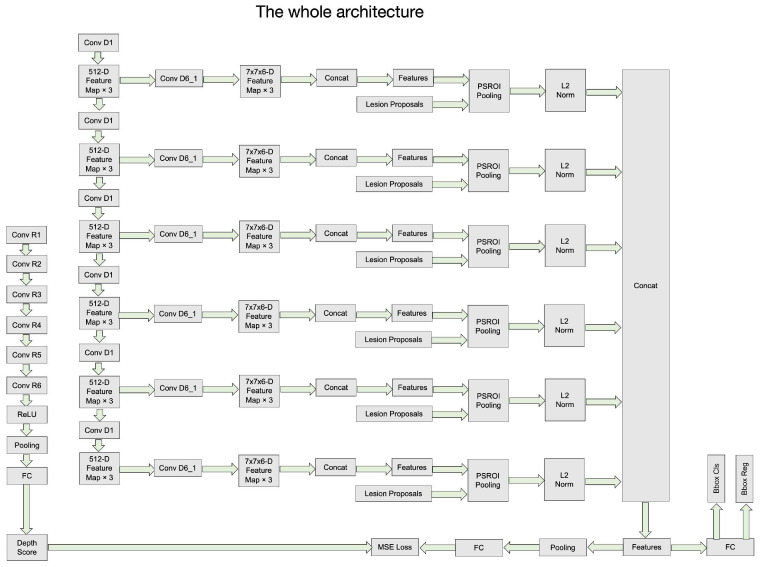
The whole architecture of our DA-SHT Dense 3DCE Network, using our Novel Dense 3DCE R-FCN as the backbone framework.

**Figure 4:**
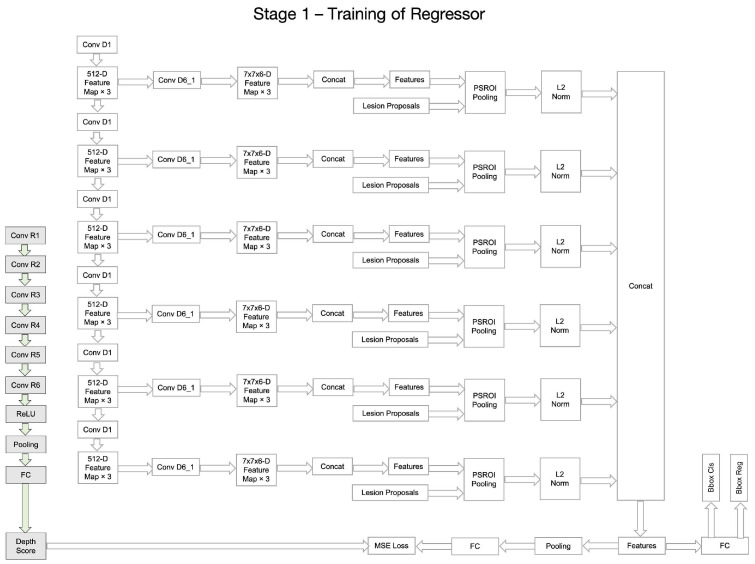
The regressor training process of our DA-SHT Dense 3DCE Network, using our Novel Dense 3DCE R-FCN as the backbone framework. The training of the regressor and the detector are consecutive steps. The regressor is trained first, and then the detector is trained.

**Figure 5:**
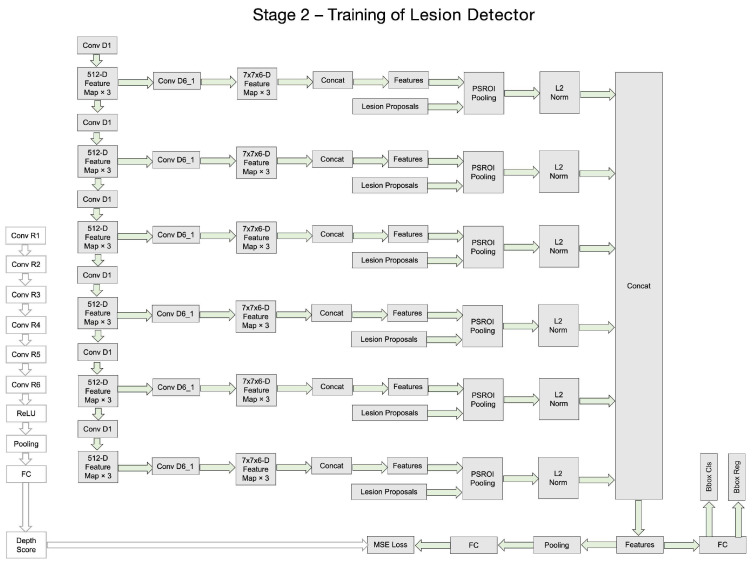
The lesion detector training process of our DA-SHT Dense 3DCE Network, using our Novel Dense 3DCE R-FCN as the backbone framework. The training of the regressor and the detector are consecutive steps. The regressor is trained first, and then the detector is trained.

**Figure 6:**
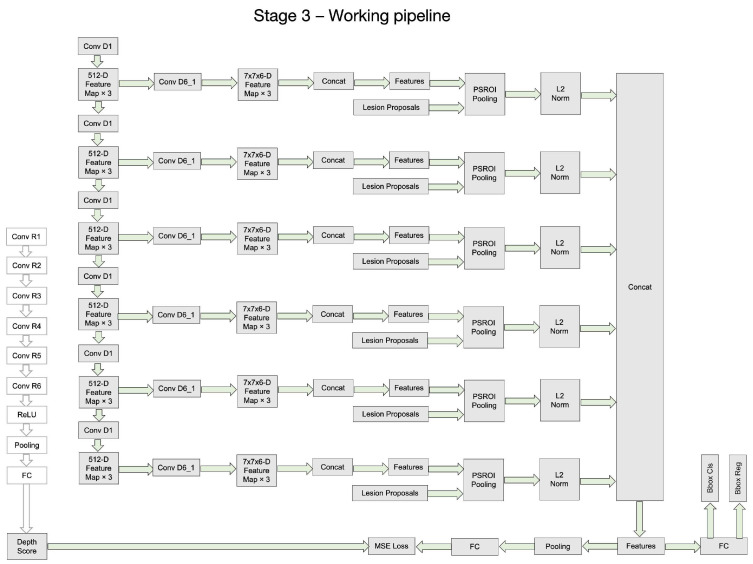
The working pipeline of our DA-SHT Dense 3DCE Network, using our Novel Dense 3DCE R-FCN as the backbone framework. The training of the regressor and the detector are consecutive steps. The regressor is trained first, and then the detector is trained.

Figure 3, Figure 4, Figure 5 and Figure 6 provide a stage-by-stage illustration of the proposed architecture, organized along a time axis to depict both parallel and consecutive steps. The process begins with the depth score regression, which is followed by lesion detection. Next, the architecture performs model training and inference, where the final predictions are made based on the predicted depth scores. The training pipeline is shown in Figure 6. This comprehensive depiction allows for a clear understanding of the workflow, highlighting the interaction between each stage and the flow of information from start to finish.

As shown in Figure 2, the entire framework consists of two sequential stages: the depth score regressor and the lesion detector. As illustrated on the left side of Figure 2, the initial stage involves an unsupervised regressor that generates depth scores for the CT slices, serving as a pre-processing method. The regressor is first trained to produce these depth scores, which are then fixed and utilized in the subsequent lesion detection stage, as indicated by the arrow pointing to the green dotted box. In this second stage, the detection network is trained to identify lesion bounding boxes. The individual blocks and layers of the depth score regressor, the pre-trained model and the lesion detector can be found in Table 1, Table 2 and Table 3 respectively.

In the depth score regressor, the starting slice *j* and the slice interval *k* of the input slices are determined randomly. The network’s layers include convolution, rectified linear unit (ReLU), and max pooling, with parameters adopted from ImageNet pre-trained CNN models, such as AlexNet [38] or VGG-16 [20]. After these layers, a new convolutional layer, Conv6, with 512 1 × 1 filters and a stride of 1, followed by a ReLU layer, is added. Conv1–Conv6 are used to learn discriminative deep image features for depth score regression. Subsequently, a global average pooling layer summarizes each of the 512 activation maps to one value, resulting in a 512D feature vector. Finally, a fully connected (FC) layer projects the feature vector to the slice score. The individual blocks and layers of the depth score regressor can be found in Table 1.

As for the lesion detector on the right side of Figure 2, every three images are treated as a 3-channel image (one sample). These images serve as input for the feature extraction network. During training, the central image provides bounding box location information, while the other slices provide 3D context information [1].

Following the 3DCE network, we adopt the novel Dense 3DCE R-FCN model [3], which extracts feature maps at various scales from the Conv1 to Conv5 layers. Each convolution block in the feature extraction network (Conv1 to Conv5) has 64, 128, 256, 512, and 512 filters, respectively. The pathways of Conv2 and Conv4 are omitted since they are reduplicative. These feature maps are then processed through a convolutional layer for dimension reduction, depicted as a series of Conv6 layers in Figure 2. A reshaping operation concatenates the feature maps, generating 3D context information. This is followed by a PSROI pooling layer with a filter size of 7×7. To normalize the feature maps, an L2 norm layer is applied after the PSROI pooling layer [39], ensuring all level features are obtained. Finally, another concatenation operation combines these features, and the fully connected layers at the end of the pipeline generate the final prediction results. The individual blocks and layers of the pre-trained model and the lesion detector can be found in Table 2 and Table 3.

The gray RPN block is designed to generate proposals. The RPN subnetwork processes feature maps extracted from the central images containing ground-truth information. Ultimately, the detection pathway on the right produces the final classification results and detection bounding boxes. The blue and red dotted boxes represent the original and auxiliary losses, respectively, with their functions detailed in the subsequent sections.

### 3.1. Novel Dense 3DCE R-FCN

In CT slices, the lesion sizes can vary significantly. For example, the long diameters of lesions in the DeepLesion dataset range from 0.42 mm to 342.5 mm, while the short diameters range from 0.21 mm to 212.4 mm [22]. The smallest lesion is nearly 1000 times smaller than the largest lesion. Such great differences in lesion sizes can bring significant challenges to lesion detection tasks and result in a high rate of false positives (FPs). Thus, we attempt to utilize multi-level and multi-resolution feature maps and generate a dense connection mechanism to meet the needs of both ULD and MOLD tasks in a dataset containing various large and small lesions.

We adopt the 3DCE R-FCN model as the backbone of our framework, as mentioned in Section 2.3. The 3DCE R-FCN network is formulated with reference to the original R-FCN model [21] but includes four additional layers, including one fully connected (FC) layer, one rectified linear unit (ReLU) activation layer, and two FC layers for the final prediction results. Compared with the Faster R-CNN model, the R-FCN network can utilize the position information of a CT slice image through a PSROI pooling layer. We use the VGG-16 CNN model [20] as the feature extractor, just as described in [1], removing the 4th and 5th pooling layers to maintain the resolution of the feature maps and prevent the feature maps from becoming too small. As shown in Figure 2, in the second stage, every three images can be viewed as a 3-channel image (one sample). Then, these three images are used as the input for the feature extraction network. During training, the central image provides the bounding box location information, and the other slices offer the 3D context information [1]. For each convolution block in the feature extraction network from Conv1 to Conv5, the number of filters is 64, 128, 256, 512, and 512, respectively. The RPN subnetwork only accepts the feature maps extracted from the central images containing the ground-truth information.

CNNs always convolve and pool images to extract more discriminate features, which are more suitable for large rather than small lesion detection, but the deeper the feature extractor is, the smaller the resolution the feature maps have. These smaller feature maps can challenge the detection of small lesions, which only occupy a few pixels. Thus, the necessity to fully use feature maps of various resolution scales is significant. Based on the 3DCE network, we follow the novel Dense 3DCE R-FCN model [3], which extracts feature maps in various scales from the Conv1 layer to the Conv5 layer for an extension, as shown in the second stage in Figure 2. These feature maps integrate the image features, which are shallow but high in resolution, intermediate but complementary, and deep but contain rich semantic information [40]. All these feature maps are then delivered to one convolutional layer for dimension reduction, which can be seen as a series of Conv6 layers in Figure 2. After that, a reshaping operation concatenates the feature maps together and generates 3D context information. Then, one PSROI pooling layer follows, and the filter size is 7×7. In order to normalize the feature maps in different amplitudes, an L2 norm layer is used after the PSROI pooling layer [39]. Therefore, all level features are obtained. Finally, another concatenation operation is used to combine all-level features. Meanwhile, the fully connected layers work at the end of the pipeline to obtain the final prediction results.

To provide a clearer comparison between our proposed model and the 3DCE R-FCN model described in [1], the framework architecture of 3DCE R-FCN is illustrated in Figure S1 of the Supplementary Materials. As depicted in Figure S1, the primary distinction from our proposed method lies in the fact that the 3DCE R-FCN model employs a one-stage end-to-end training architecture. Additionally, it does not incorporate dense pathways or auxiliary losses, as highlighted in the dotted red boxes in Figure 2.

### 3.2. Skipped-Layer Hierarchical Training

**Figure 7:**
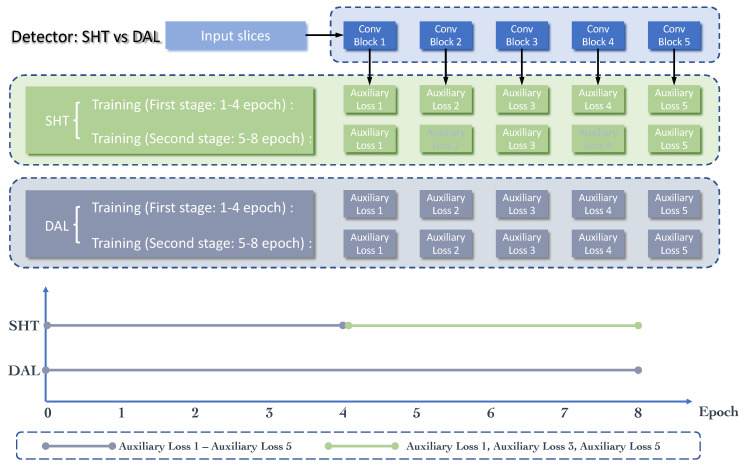
Schematic diagram comparing our proposed SHT scheme and the DAL scheme proposed in [3]. The main difference is the auxiliary loss selection in the 5-8 epoch during the training process.

The employment of the dense connections described in Section 3.1 can easily cause gradient vanishing and model degradation [36] because the depth and width of the model are both increased. Thus, we also need to overcome these limitations. An effective method is to strengthen the supervision of the middle layers. As shown in Figure 2, in the second stage, the method presented by Zhang et al. [3] employs some auxiliary losses after each pooled 3D context feature map, which can be seen from the red dotted boxes in Figure 2, as well as the gray part (gray “Auxiliary Loss 2” and “Auxiliary Loss 4”) in Figure 7. Rather than only having the classification and regression losses at the output layer, these auxiliary losses can further provide integrated optimization via direct supervision of the earlier hidden layers (from Conv1 to Conv5). Furthermore, these “auxiliary losses” can speed up the network’s convergence through their supervision pathways [36]. From [3], it is clear that through these additional pathways, the model is also forced to learn adequate discriminate features from the shallow layers, which can boost the detection performance, especially on the small lesions.

However, utilizing all these auxiliary losses in the whole training process is not an optimal training strategy. The training process of the CNN model is an iterative optimization process to find the most suitable fitting model. In the early stages of network training, the network focuses on coarse-grained learning, while in the later stages, fine-grained learning gradually becomes the emphasis of this training process. Therefore, we improve the DAL strategy in [3] to the SHT strategy, achieving optimal performance. As shown in Figure 7, in the first stage of training, we adopt all the auxiliary losses, which can be noted as dense auxiliary losses in [3]. Then, only skipped-layer losses from Conv1, Conv3, and Conv5 pathways are retained in the second stage of training. This kind of hierarchical training strategy can reduce over-fitting to some extent.

We minimize the objective functions following the multi-task loss in Faster R-CNN [20]. These losses refer to the classification loss ($L_{cls}$), the regression loss ($L_{reg}$), and the depth-aware loss ($L_{depth}$), which is highlighted in the green dotted box in Figure 2. The classification loss ($L_{cls}$) and the regression loss ($L_{reg}$) consists of two parts. The first part is the auxiliary losses, which are highlighted inside the red dotted boxes in Figure 2, while the second part is the original classification and regression losses as in [1], which are located at the end of the whole framework and are highlighted inside the blue dotted boxes in Figure 2. These losses are not combined in the network but jointly optimized through the backpropagation process during training. In Equation (1) (Note: Equations (1) to (7) are all described for the MOLD task, which includes the background class and eight lesion classes, but not the ULD task):

(1) $$L=L_{cls}+\alpha L_{reg}+\beta L_{depth},$$

here, as shown in Equation (1), the loss function contains three components: the classification loss ($L_{cls}$), the bounding box regression loss ($L_{reg}$) and the normalized depth score regression loss ($L_{depth}$). In the training process, we set α=10 and β=1 to control the relative importance among these three components. The details of $L_{depth}$ are described in Section 3.3.2).

(2) $$L_{cls}=L_{cls\_rpn}+L_{cls\_det},$$

(3) $$L_{cls\_rpn}=\frac{1}{N_{cls\_rpn}}\sum_{i\epsilon I}-log\left(\left[{\hat{p}}_{i}p_{i}+\left(1-{\hat{p}}_{i}\right)\left(1-p_{i}\right)\right]\right).$$

As shown in Equation (2), the classification loss $\left(L_{cls}\right)$ consists of two components, the RPN classification loss $\left(L_{cls\_rpn}\right)$ and the detection classification loss $\left(L_{cls\_det}\right)$. In Equation (3), we set $N_{cls\_rpn}$ = 256 because, in the training process of the RPN sub-network, we selected 256 anchors in a mini-batch. *i* is the index of the anchors in the mini-batch. *I* is the anchor set in a mini-batch. $p_{i}$ is the predicted probability of the *i*-th anchor being the foreground object, while ${\hat{p}}_{i}$ donates the ground-truth label of the anchor (1 for positive and 0 for negative).

(4) $$L_{cls\_det}=\frac{1}{N_{cls\_det}}\sum_{i\epsilon I}\sum_{d\epsilon D}\sum_{c\epsilon C}-log\left(\left[{\hat{p}}_{cid}p_{cid}\right]\right).$$

In Equation (4), the $L_{cls\_det}$ term is normalized by the mini-batch size (i.e., $N_{cls\_det}$ = 256). Unlike the classification loss in the RPN sub-network, which is a binary cross-entropy loss, the classification loss in the detection network is a multi-class cross-entropy loss. *d* is the index of the supervision pathway, where D=[1,2,3,4,5] in the first 4 training epochs, which indicates that there are a total of 5 pathways that take effect in the first training stage. Then, we set D=[1,3,5] in the following 4 epochs since the second and fourth pathways have been removed. *c* represents the class of the lesions in the dataset, and *C* is [0,8], which means that there is a total of 9 classes, including the background class. Other parameters, which are not mentioned here, are the same as those in Equation (3).

(5) $$L_{reg}=L_{reg\_rpn}+L_{reg\_det},$$

(6) $$L_{reg}=\left[{\hat{p}}_{id}>0\right]\frac{1}{N_{reg}}\sum_{i\epsilon I}\sum_{d\epsilon D}\sum_{i\epsilon \{x,y,w,h\}}L_{1smooth}\left({\hat{t}}_{id}-t_{id}\right),$$

(7) $$L_{1smooth}\left({\hat{t}}_{id}-t_{id}\right)=\left\{\begin{array}{c} {\left(\sigma \left({\hat{t}}_{id}-t_{id}\right)\right)}^{2}/2,\;if\;\left|{\hat{t}}_{id}-t_{id}\right|<1/\sigma^{2},\\ |{\hat{t}}_{id}-t_{id}|-0.5/\sigma^{2},\;otherwise. \end{array}\right.$$

Similar to the classification loss, as shown in Equation (5), the regression loss ($L_{reg}$) also consists of two components, the RPN regression loss $\left(L_{reg\_rpn}\right)$ and the detection regression loss $\left(L_{reg\_det}\right)$. Both regression losses can be calculated through Equation (6). In Equation (6), $\left[{\hat{p}}_{id}>0\right]$ is an indicator function, which aims to ignore the regression loss of the background ROIs by setting the value to 1 if ${\hat{p}}_{id}>0$ and 0 otherwise. $N_{reg}$ represents the number of anchor locations. $t_{id}$ = ($t_{xd},t_{yd},t_{wd},t_{hd}$) donates the parameterized coordinates of the predicted bounding boxes of the *d*-th supervision pathway, where x,y,w and *h* denote the box’s center coordinates and its width and height, respectively. $\hat{t_{id}}$ represents the parameterized coordinates of the ground-truth box with a positive anchor. Other parameters, which are not mentioned here, are the same as those in Equation (4). The smooth $L_{1}$ loss is defined in Equation (7), and the details can be found in [25]. In the RPN training process, we set σ to 3, while in the detection process, it is set to 1, which is the same as in [1,25]. According to the explanation from the authors of [25], setting σ to 3 in the training process makes the transition point from quadratic to linear happen at |x| < $\frac{1}{9}$, which is closer to the origin. The reason for doing this is because the RPN bounding box regression targets are not normalized by their standard deviations, unlike in Fast R-CNN [24], because the statistics of the targets are constantly changing throughout learning.

### 3.3. Depth-Aware Mechanism

In practice, clinicians use long diameters and short diameters to mark the locations of the lesions [22]. In this way, they can save time because they do not need to draw curves to circle the outlines of the lesions. However, different kinds of lesions have different shapes, such as ellipses or bands. A lack of information about a lesion’s shape can increase the difficulty of classifying its type. Thus, we have to explore more discriminate information to assist the lesion type classification process. Therefore, taking domain knowledge into consideration as well as including depth information in the training process (which will be described in this section) is a good alternative to the lack of shape information and can also assist the process of classifying lesion types.

#### 3.3.1. Unsupervised Depth Score Regression

During the first stage of the whole framework, an unsupervised depth score regressor is introduced, which is the same as in [19]. The regressor can predict a continuous score for each axial CT slice, representing the normalized axial coordinates. This process is unsupervised, and no extra annotation efforts are needed. The inputs are unlabeled CT slice volumes. Although the scores of all slices can be obtained via the regressor, only the scores of the central images are used as the input of the second stage. This is because only the central slices contain the ground-truth bounding boxes and are used for the final prediction.

Regarding the input, several slices with the same distance are picked from each volume in each training iteration. As shown in Figure 2, the starting slice *j* and the slice interval *k* are determined randomly in the first stage. The order loss constrains the larger slices with larger scores and the distance loss, ensuring that the linear increase of the scores is collaborated simultaneously to constrain the regressor to follow the superior-inferior ordering rule and ensure that the distance increases linearly.

#### 3.3.2. The Depth-Aware Pathway

As shown in Figure 2, the second stage of our framework uses a multi-organ lesion detector. The depth scores generated in the first stage are delivered to this detector through the depth-aware pathway. This can be seen from the arrow pointing to the green dotted box. We can see from Figure 2 that the detector consists of two pathways followed by the final feature map: the detection pathway on the right gives the final results containing the classification results and detection bounding boxes, while the depth-aware pathway on the left (shown in the green dotted box), which aim to promote the detection results by providing depth information learned from the inter-slice relationships. The training of the regressor and the detector is not an iterative approach. The predicted depth scores from the regressor are fixed and then sent to the detector as inputs when training the detector. The depth-aware pathway can provide the location information from the 3D depth perspective, while the lesion detection pathway can extract more discriminate location features from the 2D height and width perspective. The two pathways are complementary by nature in this CT MOLD task.

For the depth-aware pathway, in order to solve the problem of different input sizes to the FC layer, a 7×7 PSROI pooling layer is added before the FC layer performs the ROI pooling operation on the entire set of images. Meanwhile, a ReLU activation function is also used after the FC layer. Loss functions, including the regression loss in the depth-aware pathway as well as the classification losses and regression losses in the detection pathway, are optimized jointly as shown in Equation (1). The depth loss ($L_{depth}$) is derived based on Equation (8).

(8) $$L_{depth}=\frac{1}{N_{depth}}\sum_{i}{\left(s_{i}-\hat{s_{i}}\right)}^{2}.$$

In $L_{depth}$, *i* is the index of the central images in a mini-batch. The term is calculated by finding the mean value of $N_{depth}$, which is the size of the mini-batch. $s_{i}$ denotes the output scores of the depth score regressor in the first stage, while $\hat{s_{i}}$ denotes the predicted scores in the depth-aware pathway of the second stage. We use the mean squared error (MSE) loss to provide the depth information in the backpropagation process.

The estimated depth information can provide useful 3D spatial information for each voxel, and the feature maps extracted from the 2D CT images can provide more fine-grained textual information. These two pieces of information are complementary by nature. Rather than using 3D pixel volumes as input to extract the depth information, the DA-SHT Dense 3DCE model uses the 2D CT slice images as input. Then, the unsupervised regressor generates the depth information at the feature map level. Thus, our model significantly saves computing resources as compared with the 3D Conv-Nets.

### 3.4. Participants and Dataset

We have evaluated the proposed method on the publicly-available DeepLesion dataset [22], which contains 32,735 lesions from 32,120 axial CT slices from 10,594 CT scan studies of a total of 4427 patients. Different from other datasets, which contain lesions from benign and malignant categories, the DeepLesion dataset does not classify the lesions into benign or malignant. Each CT slice has 1–3 lesions with corresponding bounding boxes and size measurements. All lesions in the dataset have been annotated using the REICIST diameters, including the long and short diameters. The resolution of most of the images is 512×512, while only 0.12% of the images have a resolution of 768×768 or 1024×1024. To investigate the lesion types in DeepLesion, the authors in [22] randomly chose 9816 lesions and manually categorized them into eight categories. There are a total of 8 different types of lesions with different proportions in the 9,816 lesions, including bone (BN, 2.5%), abdomen (AB, 22.1%), mediastinum (ME, 17.0%), liver (LV, 13.0%), lung (LU, 24.3%), kidney (KD, 5.0%), soft tissue (ST, 6.9%) and pelvis (PV, 8.8%) [18]. The mediastinum lesions are mainly composed of tibial lymph nodes [22]. Abdomen lesions are complications other than the liver or kidneys. Soft tissue types included muscle, skin, and minor complications [22].

In order to better evaluate the performance of the proposed method, we extracted one small lesion dataset and one multi-organ lesion dataset from the original DeepLesion dataset. We selected the small lesions whose areas were less than 1% of the largest lesion to build a small lesion dataset. We extracted the multi-organ lesion dataset because only 30% of the samples of the DeepLesion dataset were given category information. Therefore, only 9816 lesions on 9626 axial slices were used for comparison experiments. The statistical details of the official DeepLesion dataset and its subsets can be found in Table 4. We focused on the ULD task on the original DeepLesion dataset and the small lesion dataset, which only distinguishes lesions from non-lesions. We focused on the MOLD task for the multi-organ lesion dataset, which provides bounding boxes with specific lesion type results. Our method’s effectiveness can be further investigated by testing it on these three datasets.

### 3.5. Procedure

**Figure 8:**
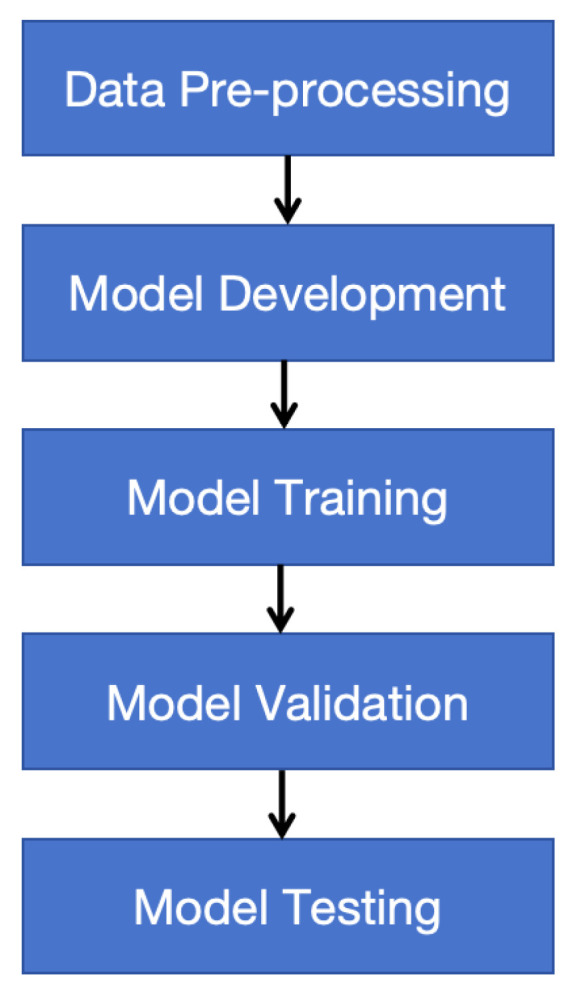
This flowchart illustrates the procedural steps followed in our study. The steps include data pre-processing, model development, model training, validation, and testing.

The procedure of our study is illustrated in Figure 8. The steps are as follows:

- Data pre-processing: The CT scans were preprocessed to normalize the pixel intensities and resize the images to a uniform resolution.
- Model development: We developed the Dense 3D context-enhanced (Dense 3DCE) network, integrating depth-aware (DA) and skipped-layer hierarchical training (SHT) mechanisms.
- Model training: The model was trained on the DeepLesion dataset, using a combination of supervised learning techniques to optimize the detection accuracy.
- Model validation: We validated the model using the original and the selected small dataset to ensure generalizability and robustness.
- Model testing: The model’s performance was tested on a separate subset of the DeepLesion dataset to evaluate its effectiveness in detecting lesions of varying sizes and appearances.

### 3.6. Instruments and Implementation Details

We carried out all our comparison experiments with MXNet [41] 1.5.0 on a PC equipped with one NVIDIA 2080 GPU. To ensure a fair comparison, we configured all hyperparameters in accordance with the settings used in the 3DCE R-FCN network [1]. We used the stochastic gradient descent (SGD) method with a momentum value of 0.9 to be the optimizer of the model. We set the initial learning rate at 0.001 and decreased it by 10 times after the 4th, 5th, 7th, and 8th epochs. In order to make a fair comparison, all models were trained for 8 epochs. Due to the limited GPU memory, the batch size could only be set to 1 in the training process. Meanwhile, we used three ratios (1:1, 1:2, 2:1) and five scales (16, 24, 32, 48, 96) to generate the anchors.

### 3.7. Pre-Processing

For pre-processing, we followed the method presented in [1] and used intensity windowing to rescale the images to ensure that the intensity range became [0, 255] in the floating-point number format. The black border was also split. Meanwhile, to make sure that each pixel corresponded to 0.8mm, we also rescaled the images as the inputs to the network. Most CT scans in the dataset have either 1 mm or 5 mm slice intervals. At the same time, through image interpolation along the z-axis, we made the intervals of all volumes the same (2 mm). When doing experiments on the original DeepLesion dataset and the small lesion dataset, we used the official data split provided by the DeepLesion dataset, which included 70% samples for training, 15% for validation, and 15% for testing. Regarding the multi-organ dataset, we could only use a random split with the same ratio among the training (70%), validation (15%), and testing (15%) sets because an official data split was unavailable. We also made sure that there was no data leakage between training, validation, and testing datasets for the multi-organ dataset. To compare the methods fairly, we used the same data split in all experiments on all three datasets (the original DeepLesion dataset, the small lesion dataset, and the multi-organ lesion dataset). We do not conduct any data augmentation on the dataset. Note that for the bounding box predictions, if an intersection over union (IoU) with the given ground-truth bounding boxes was larger than 0.5, it was declared correct; otherwise, it was declared negative.

### 3.8. Data Analysis

To compare the performance of different methods quantitatively, we utilized the average sensitivity (AS) and mean average precision (mAP) values as the evaluation metrics. Sensitivity is a widely-used evaluation metric in the lesion detection field [1,42], while mAP is widely used in the object detection field [21,25]. The combination of these two evaluation metrics is practical in reflecting the effectiveness of the proposed method. AS and mAP were computed using the average value of over 8 lesion types. For AS, we studied the sensitivities at 6 different values [0.5, 1, 2, 4, 8, and 16] of FPs per image to compare the performance of different model variants.

We also reported the standard deviation (std) results for all reported performance metrics. To eliminate randomness in experimental results, we conducted repeated experiments on both the ULD and MOLD tasks. Specifically, for each trained model, we conducted multiple test experiments. For each test, we randomly selected 80% of the samples from the entire test set and computed the standard deviation from the results multiple times.

## 4. Experimental Results

### 4.1. Universal Lesion Detection

To evaluate the proposed DA-SHT Dense 3DCE R-FCN model, we performed extensive experiments on a publicly available DeepLesion dataset and the extracted small lesion dataset using the data split officially provided. It is obvious that among all the models, our DA-SHT Dense 3DCE R-FCN model shows the best performance on both the original DeepLesion dataset and the extracted small lesion dataset using the data split officially provided by the DeepLesion dataset. Some quantitative results for ULD on the original DeepLesion dataset and the extracted small lesion dataset are listed in Table 5 and Table 6, respectively. It is noticeable that the DA-SHT Dense 3DCE R-FCN architecture shows the best sensitivity for nearly all FP values on the original DeepLesion dataset. This indicates the effectiveness of the SHT training strategy. Meanwhile, our model also performs well on the extracted small lesion dataset, indicating a more convincing clinical value. Compared to 3DCE R-FCN, our model can improve the sensitivity by 1.53–2.19% at different FP values per image. It is noticeable that the inference time of our DA-SHT Dense 3DCE network is competitive with the 3DCE model on both datasets. This means the time cost for the boost of the performance is minimal.

However, we also note from Table 6 that the detection performance improvement of our proposed DA-SHT Dense 3DCE method is marginal compared to the Dense DAL 3DCE R-FCN [3] on the original DeepLesion dataset ULD task. A reasonable explanation for this is two-fold, including the inapplicability of the DA mechanism and the effect of SHT being diluted. First, the proposed DA mechanism provides depth information for better supervising the classification of lesion categories. However, the ULD task is essentially a binary task, which only distinguishes lesions from non-lesions. This task focuses on accurately locating bounding boxes without the need to classify lesion categories. Therefore, the effectiveness of DA is weakened here. This reason can also be evident in the ablation study section (Section 4.3). Second, besides the DA mechanism, we replace the DAL scheme with the SHT scheme in our method. This novel SHT scheme produces good results mainly for small lesions, which can be proved from Table 5. However, the proportion of the small lesions in the original DeepLesion dataset is relatively low, which causes the improvement not to be significant when we perform the ULD task on the original DeepLeison dataset. This can be seen as a dilution of the detection performance boost on the small lesions.

Nevertheless, our work makes significant contributions to front-line screening universal lesion detection CADx systems. Future evaluations of disease-specific lesion detection CADx systems should compare not only against other disease-specific systems [43,44,45,46,47] but also against universal lesion detection CADx systems [48].

### 4.2. Multi-Organ Lesion Detection

The evaluation presented in this section mainly focuses on the MOLD task so as to better prove the clinical value of our CADx system. Therefore, we have evaluated the effectiveness of our overall model by using the multi-organ lesion dataset, which was extracted from the original DeepLesion dataset. As listed in Table 7, we compare our model with six baseline methods, the faster region-based CNN (Faster R-CNN), the original R-FCN, the 3DCE network, Dense DAL 3DCE R-FCN, Dense DKS 3DCE R-FCN, and MP3D model. The Faster R-CNN and the original R-FCN network only use three neighboring slices, and no feature map fusion is used. For a fair comparison and limited by the GPU memory, we used 9 slices in the 3DCE, Dense DAL 3DCE R-FCN, Dense DKS 3DCE R-FCN, MP3D network, and DA-SHT 3DCE models. We set the depth of the MP3D model to 18 because of the limited GPU memory. The number of stages is set to 2, and the data augmentation training scheme and the COCO dataset [49] pre-trained model are both removed for a fair comparison. In Table 7, it is demonstrated that our DA-SHT 3DCE model has convincing results in both the AS and mAP metrics. Compared with two widely-used baseline methods, Faster R-CNN, and the original R-FCN, it boosts the performance by around 6.19–17.60% on AS at various FPs per image and 8.19–14.65% (average 11.43%) on mAP. Compared to the 3DCE network, Dense DAL 3DCE R-FCN, Dense DKS 3DCE R-FCN, our DA-SHT 3DCE model constantly improves the MOLD results by 1.72–15.21% in AS and 3.63–10.63% in mAP. As for the MP3D model, our method improves the sensitivities at different false positive values by 0.05–5.72%.

In total, there are eight different types of lesions in the DeepLesion dataset. In order to better evaluate the sensitivity of the network to different kinds of lesions, we have also analyzed the detection accuracy of different lesion types. The abbreviations of eight lesion type names are mentioned in Section 3.4. In order to intuitively show the performance of all methods for different lesions, we use bar charts to illustrate the results. Table 8 shows the sensitivity at 4 FPs per image on different lesions. The results of our network surpass baseline methods in most lesion types with a convincing margin. For the most common types of lesions, those in the lungs and abdomen, our DA-SHT 3DCE model increases the sensitivity by around 1.42–15.65%. In particular, for infrequent bone lesions, which only occupy 2.5 % of the entire dataset, our model achieves a significant increase of at least 5.41% when compared with the other baseline models. This indicates that our model can give a good detection performance even when the data is limited. Similar to the trend in Table 8, the AP values in Table 9 also show a similar trend on different lesions.

**Figure 9:**
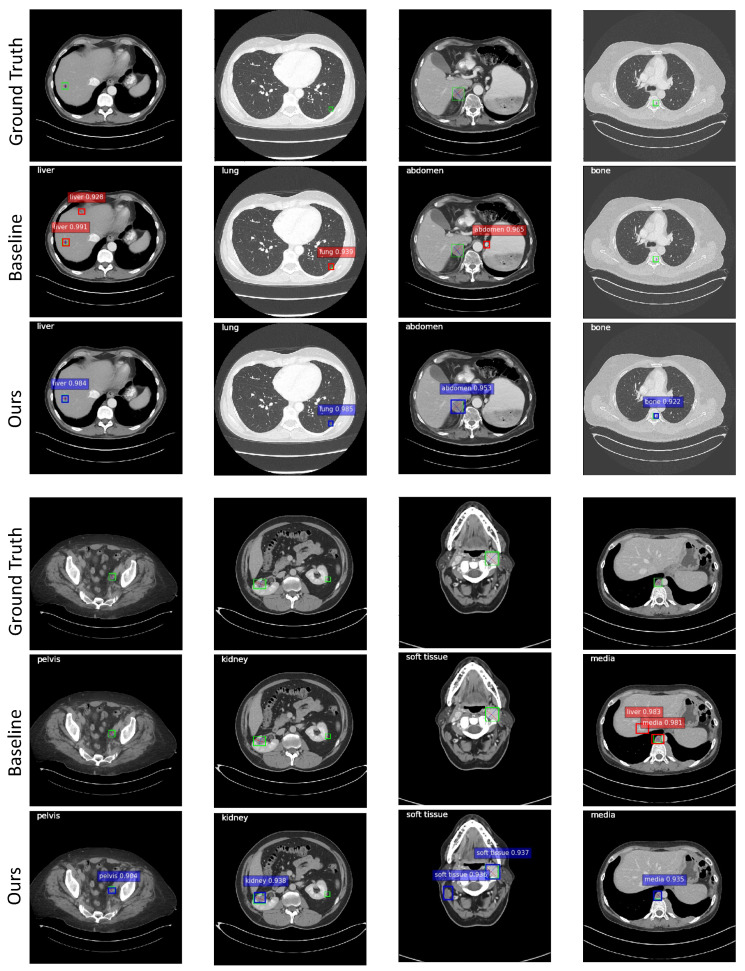
Qualitative results using the DA-SHT Dense 3DCE framework on different lesions. The images in the second and fifth rows show the results of the 3DCE [1] network, and the images in the third and sixth rows show the results of our model. Predictions with scores >0.9 are shown. The categorical information in the upper left corner of each image is the ground truth of the lesion. The green bounding boxes with diameters in the first and fourth rows are ground truth bounding boxes. The bounding boxes in red and blue highlight the categorical and confidence scores of the automatic detection results, respectively.

**Figure 10:**
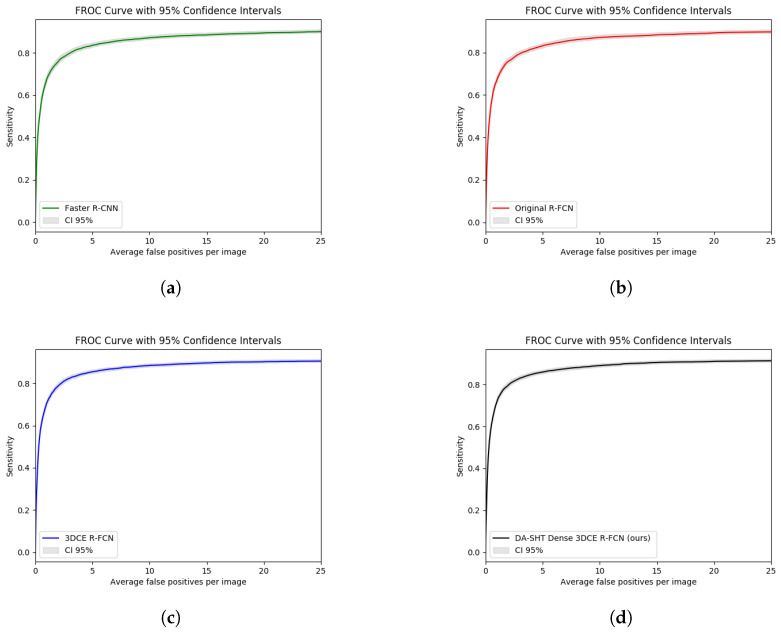
FROC curves of our proposed and baseline methods used for the ULD task on the original DeepLesion dataset. (**a**) Faster R-CNN; (**b**) Original R-FCN; (**c**) 3DCE R-FCN; (**d**) DA-SHT Dense 3DCE R-FCN (Ours).

Figure 9 illustrates some qualitative results. The images in the second and fifth rows show the results of the 3DCE [1] network, and the images in the third and sixth rows show the results of our model. Predictions with scores >0.9 are shown. Predictions with scores >0.7 and >0.8 can be found in Figures S2 and S3 in the Supplementary Materials. The categorical information in the upper left corner of each image is the ground truth of the lesion. The green bounding boxes with diameters in the first and fourth rows are ground truth bounding boxes. It can be observed from the samples that DA-SHT 3DCE R-FCN can effectively reduce FPs and FNs, and it can give higher confidence scores for true positive results. The cases can also identify the depth-aware mechanism that can reduce the misclassification errors in the MOLD task to some extent. In order to provide a more intuitive and rigorous quantitative comparison, we also used the free-response receiver operating characteristic (FROC) curves with 95% confidence intervals (CI95) to evaluate the performance of our method and the selected baseline method, as shown in Figure 10. We also provide comparison results of the baseline and proposed modules during the training and analysis steps, which can be found in Table 10. We report the validation AS value at 4 FPs per image during the whole training process.

### 4.3. Ablation Study

In order to better analyze the contributions of the three schemes, the multiple resolution feature map pathways (“DENSE” in Table 11), the SHT strategy (“SHT” in Table 11), and the depth-aware scheme (“DA” in Table 11), the distinct impacts of our proposed additions mentioned above, which result in our final formulation DA-SHT 3DCE R-FCN have been investigated. In the ablation study experiments, first, we evaluated the 3DCE backbone [1] with default settings, and after that, we incrementally added the three schemes as mentioned above. Since the inclusion of the SHT strategy was implemented based on the dense connection mechanism, we omitted the combination of 3DCE + SHT. Table 11 summarizes these results. 3DCE denotes the backbone, which was first proposed in [1]. DENSE denotes using feature maps in all resolutions, which was introduced in Section 3.1. DAL, SHT, and RANDOM all refer to the strategy by which we select the auxiliary losses during the training process. They are contrary to each other. Therefore, they cannot appear in the same network. The details can be found in the caption of Table 11. DA denotes the depth-aware scheme we proposed and described in Section 3.3.

Regarding the ULD task, the effectiveness of SHT can be found in the last three rows of Table 11. After employing the DENSE+SHT+DA algorithm to optimize the training process, our DA-SHT 3DCE already outperforms the DENSE+DAL+DA and DENSE+RANDOM+DA combinations. The sensitivity at 2.0 FPs is 0.15% higher than with DENSE+DAL+DA and 0.10% higher than with DENSE+DAL+DA at 4.0 FPs. The possible explanation of the marginal boost is two-fold, containing the DA mechanism’s inapplicability and the effect of SHT being diluted, which has already been analyzed in Section 4.1. As for the DA scheme, the original 3DCE + DA scheme performs poorly on the ULD task, indicating that only using the depth information from the CT slices is sub-optimal in the ULD task. This is because the depth information contributes to the classification process. However, the ULD only distinguishes lesions from non-lesions, which is not challenging in the classification process, making the DA mechanism’s effectiveness indemonstrable. In addition to this, the predicted depth scores are not fully accurate as they are outputs from an unsupervised network. Therefore, adding the DA scheme when conducting the ULD task means that the network has to fit the depth scores, which are less relevant to the ULD task and contain noise. As a result, the DA scheme can interfere with the network and lead to poor performance.

We have also carried out a complete series of ablation studies on the MOLD task. From Table 11, we can see that the proposed three mechanisms contribute more to this MOLD task than the ULD task. From the first and second row of Table 11, it is obvious that adding the DA mechanism significantly boosts the detection sensitivity of all FP rates, with an improvement of 3.22% at 2.0 FPs, 2.24% improvement at 4.0 FPs, and 3.31% improvement at mAP. Moreover, with the proposed mechanism of SHT to replace the original DAL, the sensitivity can be further improved from 66.25% to 67.29% (2.0 FPs per image), which can be attributed to the SHT mechanism. From the last three rows of Table 11, the effectiveness of reducing part of the auxiliary losses has been proved. Although keeping the 1st, 3rd, and 5th auxiliary losses can achieve the best performance, selecting three auxiliary losses randomly can still improve the detection performance compared to using all auxiliary losses. This hierarchical training strategy can make the losses more adaptive to the task we worked on and reduce over-fitting as well as the number of parameters. The depth-aware scheme also adds significant training signals, providing more accurate localization information except for the 2D bounding boxes. Consistency between the z-axis depth score and the lesion type classification helps to reduce FPs. It is obvious that these three mechanisms can further boost the performance, which is proved by the sensitivity of 73.88% at 4.0 FPs, the improvement of almost 10% at 2.0 FPs, and almost 8 % in mAP value over the best-reported results of the baseline 3DCE model.

### 4.4. Sensitivities to Hyper-Parameters

There are multiple hyper-parameters between losses, e.g., α and β in Equation (1). In Table 12, we investigate the settings of the hyper-parameters α and β. By default, we fixed α = 10 according to [25] and set β = 1, which makes the three terms in Equation (1) roughly equally weighted after normalization. For better comparison, we conducted the comparison experiments on different β values on a scale of about three orders of magnitude (0.1 to 100). From the results in Table 12, it is obvious that the choice of hyper-parameter β is sensitive to the final detection results. When setting β = 0.1, 10, 100, the sensitivity at 2 FPs drops by a considerable margin of 7–9% compared to the default setting (β = 1), while the mAP decreases following the same trend, which is 6–8%. To get the best performance, we fixed β to 1 as mentioned in Section 3.2.

### 4.5. Analysis of the Predicted Depth Scores

The predicted depth scores from the first stage regressor may not always be accurate enough, directly affecting the training process and inference results of the second stage detector. To evaluate how the inaccurate depth scores predicted by the regressor affect the subsequent detection tasks, we added different levels of Gaussian noise to the depth scores predicted from the first stage and utilized these depth scores for the second stage training process. The comparison experiments are shown in Table 13. It is obvious that the detector is not very sensitive to the noise in the depth scores. In addition, the experimental results also demonstrate that the final detection results are robust to different noise levels. A reasonable explanation is that organs are three-dimensional structures occupying a specific depth range. Therefore, weak noise on depth scores will not significantly impact the category classification results.

## 5. Discussion

The results of our study indicate that the proposed DA-SHT Dense 3DCE network significantly enhances the performance of multi-organ lesion detection (MOLD) in chest CT scans. Our findings are consistent with several key studies in the literature, yet also provide unique contributions to the field.

First, our approach outperforms the original 3DCE network, as demonstrated by the superior detection accuracy for both large and small lesions. This aligns with the findings of [3], who also emphasized the importance of incorporating multi-scale features for effective lesion detection. However, our study extends their work by introducing depth-aware (DA) mechanisms, which significantly improve the exploitation of 3D contextual information from CT volumes.

In comparison to the work of [1], who utilized ConvNets for lesion detection, our method provides a more comprehensive framework by integrating skipped-layer hierarchical training (SHT). This novel mechanism enhances the backpropagation process, ensuring that both shallow and deep layers receive adequate supervision. This contrasts with Yan et al.’s approach [1], which primarily focused on feature extraction at a single scale.

Moreover, our results highlight the effectiveness of the DA scheme in reducing common misjudgments associated with normalized CT scan depth scores. Previous studies, such as those by [50], have shown the potential benefits of depth information in medical imaging. Our work builds on these insights by demonstrating a concrete implementation within a multi-organ lesion detection framework, thereby providing a more robust model that leverages depth domain knowledge effectively.

Overall, our study confirms the value of integrating advanced mechanisms such as depth awareness and hierarchical training into lesion detection models. These innovations address several limitations identified in previous research, providing a more accurate and robust solution for multi-organ lesion detection in chest CT scans. Future research could further explore these mechanisms in other medical imaging applications, potentially leading to broader advancements in the field.

## 6. Conclusions

In this work, we have introduced a novel architecture for the MOLD task in chest CT scans, demonstrating the efficacy of integrating a SHT mechanism into our proposed Dense 3DCE network. This innovation not only retains the benefits of dense auxiliary losses—which bolster the supervision of early, shallow layers—but also encourages learning more discriminative features essential for accurate lesion detection.

The introduction of a depth score regressor into our Dense 3DCE framework allows the model to utilize 3D contextual features along with precise depth information from CT slices, enhancing our ability to exploit the intrinsic structural information of CT volumes. This approach helps in circumventing common misjudgments associated with normalized CT scan depth scores, thereby improving the detection accuracy.

### 6.1. Limitations

Despite the promising results, our study has certain limitations that must be acknowledged. First, the DeepLesion dataset, although extensive, may not represent the full spectrum of lesion types and variations seen in clinical practice. The sample size and diversity could impact the generalizability of our model to different populations and imaging conditions. Second, our approach relies heavily on the quality and resolution of the CT scans, which may not be consistent across all medical facilities. Additionally, while the DA-SHT Dense 3DCE network shows improved performance, the complexity and computational demands of the model might limit its scalability and application in real-time clinical settings.

### 6.2. Future Research Directions

Looking forward, the potential for further improving MOLD tasks remains vast. Future work could explore the integration of more complex attention mechanisms [51] to refine the feature extraction process, potentially increasing the sensitivity and specificity of lesion detection. Additionally, investigating the incorporation of generative adversarial networks (GANs) [52] could offer novel ways of augmenting training data, particularly for under-represented lesion types, thereby enhancing the robustness of the model against varied pathological manifestations. These directions not only promise to enhance the performance of our current model but also pave the way for broader applications in medical imaging diagnostics.

## Figures and Tables

**Table 1:** Details of the depth score regressor at each ConvNet’s layer (unit: pixel).

Layer Type			Kernel Attribute	Num of Filters
Image Input Layer				
Depth Score Regressor	Conv1	Convolutional Layer	3 × 3, stride = 1, padding = same	64
		ReLU Layer		
		Convolutional Layer	3 × 3, stride = 1, padding = same	64
		ReLU Layer		
		Max Pooling		2 × 3
	Conv2	Convolutional Layer	3 × 3, stride = 1, padding = same	128
		ReLU Layer		
		Convolutional Layer	3 × 3, stride = 1, padding = same	128
		ReLU Layer		
		Max Pooling		2 × 3
	Conv3	Convolutional Layer	3 × 3, stride = 1, padding = same	256
		ReLU Layer		
		Convolutional Layer	3 × 3, stride = 1, padding = same	256
		ReLU Layer		
		Convolutional Layer	3 × 3, stride = 1, padding = same	256
		ReLU Layer		
		Max Pooling		2 × 3
	Conv4	Convolutional Layer	3 × 3, stride = 1, padding = same	512
		ReLU Layer		
		Convolutional Layer	3 × 3, stride = 1, padding = same	512
		ReLU Layer		
		Convolutional Layer	3 × 3, stride = 1, padding = same	512
		ReLU Layer		
		Max Pooling		2 × 3
	Conv5	Convolutional Layer	3 × 3, stride = 1, padding = same	512
		ReLU Layer		
		Convolutional Layer	3 × 3, stride = 1, padding = same	512
		ReLU Layer		
		Convolutional Layer	3 × 3, stride = 1, padding = same	512
		ReLU Layer		
		Max Pooling		2 × 3
	Conv6	Convolutional Layer	1 × 3, stride = 1, padding = same	512
		ReLU Layer		
		Global Average Pooling		
		Fully Connected Layer		

**Table 2:** Details of the pre-train model at each ConvNet’s layer (unit: pixel).

Layer Type			Kernel Attribute	Num of Filters
Image Input Layer				
Pre-train model—VGG16 [20]	Conv1	Convolutional Layer	3 × 3, stride = 1, padding = same	64
		ReLU Layer		
		Convolutional Layer	3 × 3, stride = 1, padding = same	64
		ReLU Layer		
		Max Pooling		2 x 2
	Conv2	Convolutional Layer	3 × 3, stride = 1, padding = same	128
		ReLU Layer		
		Convolutional Layer	3 × 3, stride = 1, padding = same	128
		ReLU Layer		
		Max Pooling		2 × 2
	Conv3	Convolutional Layer	3 × 3, stride = 1, padding = same	256
		ReLU Layer		
		Convolutional Layer	3 × 3, stride = 1, padding = same	256
		ReLU Layer		
		Convolutional Layer	3 × 3, stride = 1, padding = same	256
		ReLU Layer		
		Max Pooling		2 × 2
	Conv4	Convolutional Layer	3 × 3, stride = 1, padding = same	512
		ReLU Layer		
		Convolutional Layer	3 × 3, stride = 1, padding = same	512
		ReLU Layer		
		Convolutional Layer	3 × 3, stride = 1, padding = same	512
		ReLU Layer		
		Max Pooling		2 × 2
	Conv5	Convolutional Layer	3 × 3, stride = 1, padding = same	512
		ReLU Layer		
		Convolutional Layer	3 × 3, stride = 1, padding = same	512
		ReLU Layer		
		Convolutional Layer	3 × 3, stride = 1, padding = same	512
		ReLU Layer		
		Max Pooling		2 × 2

**Table 3:** Details of the unsupervised lesion detector at each ConvNet’s layer (unit: pixel).

Layer Type			Kernel Attribute	Num of Filters
Image Input Layer				
Lesion Detector	Conv1	Convolutional Layer	3 × 3, stride = 1, padding = same	64
		ReLU Layer		
		Convolutional Layer	3 × 3, stride = 1, padding = same	64
		ReLU Layer		
		Max Pooling		2 × 2
	Conv2	Convolutional Layer	3 × 3, stride = 1, padding = same	128
		ReLU Layer		
		Convolutional Layer	3 × 3, stride = 1, padding = same	128
		ReLU Layer		
		Max Pooling		2 × 2
	Conv3	Convolutional Layer	3 × 3, stride = 1, padding = same	256
		ReLU Layer		
Lesion Detector	Conv3	Convolutional Layer	3 × 3, stride = 1, padding = same	256
		ReLU Layer		
		Convolutional Layer	3 × 3, stride = 1, padding = same	256
		ReLU Layer		
		Max Pooling		2 × 2
	Conv4	Convolutional Layer	3 × 3, stride = 1, padding = same	512
		ReLU Layer		
		Convolutional Layer	3 × 3, stride = 1, padding = same	512
		ReLU Layer		
		Convolutional Layer	3 × 3, stride = 1, padding = same	512
		ReLU Layer		
	Conv5	Convolutional Layer	3 × 3, stride = 1, padding = same	512
		ReLU Layer		
		Convolutional Layer	3 ×3, stride = 1, padding = same	512
		ReLU Layer		
		Convolutional Layer	3 × 3, stride = 1, padding = same	512
		ReLU Layer		
	Conv6_1 - Conv6_5	Convolutional Layer	1 × 1, stride = 0, no padding	7 × 7 × 6
		PS ROI Pooling		7 × 7
		Fully Connected Layer		

**Table 4:** The statistical details of the official DeepLeison dataset and the subsets for the ULD and MOLD tasks.

Dataset	Task	Total Number of Lesions				Number of CT Slices
		**Train**	**Validation**	**Test**	**Total**	
Original DeepLesion dataset	ULD	22,919	4889	4927	32,120	32,735
Extracted small lesion dataset	ULD	15,921	3537	3392	22,533	22,850
Multi-organ lesion dataset	MOLD	6871	1472	1473	9626	9816

**Table 5:** Universal lesion detection (ULD) task results and inference time on the selected small DeepLesion dataset. Sensitivity (%) at various FPs per image and the mean average precision (mAP) are used as the evaluation metrics. std denotes the standard deviation value. IT denotes the inference time.

Evaluation Metric	Sen@0.5 ± std	Sen@1 ± std	Sen@2 ± std	Sen@4 ± std	Sen@8 ± std	Sen@16 ± std	mAP ± std	IT(ms) ± std
Faster R-CNN [25]	58.93 ± 1.87	68.46 ± 0.18	76.69 ± 0.99	82.27 ± 1.75	85.98 ± 2.02	88.52 ± 1.88	52.80 ± 1.19	**200 ± 3.79**
Original R-FCN [21]	57.89 ± 0.64	68.69 ± 0.56	76.60 ± 1.17	82.12 ± 1.38	86.28 ± 1.73	88.61 ± 1.79	50.13 ± 1.02	222 ± 31.77
3DCE, 9 slices [1]	62.70 ± 0.71	72.20 ± 0.46	79.85 ± 1.58	84.51 ± 1.76	87.43 ± 1.73	89.67 ± 1.85	57.09 ± 1.93	239 ± 24.83
Dense DAL 3DCE R-FCN, 9 slices [3]	63.32 ± 1.10	72.79 ± 0.95	80.94 ± 2.29	85.93 ± 2.29	88.88 ± 2.50	90.82 ± 2.38	58.28 ± 0.77	235 ± 3.51
DA-SHT Dense 3DCE R-FCN, 9 slices (ours)	**64.86 ± 1.43**	**74.39 ± 1.01**	**81.41 ± 2.08**	**86.04 ± 2.38**	**89.26 ± 1.82**	**91.38 ± 1.77**	**59.22 ± 1.19**	241 ± 2.09

**Table 6:** Universal lesion detection (ULD) task results and inference time on the original DeepLesion dataset. Sensitivity (%) at various FPs per image is used as the evaluation metric. std denotes the standard deviation value. IT denotes the inference time.

Evaluation Metric	Sen@0.5 ± std	Sen@1 ± std	Sen@2 ± std	Sen@4 ± std	Sen@8 ± std	Sen@16 ± std	mAP ± std	IT(ms) ± std
Faster R-CNN [25]	56.19 ± 2.38	67.81 ± 0.80	75.98 ± 1.32	82.13 ± 0.68	86.14 ± 0.60	88.76 ± 0.57	50.98 ± 2.21	**207 ± 13.05**
Original R-FCN [21]	56.45 ± 0.14	67.55 ± 0.98	76.02 ± 0.73	81.72 ± 0.59	86.22 ± 0.67	88.58 ± 0.54	50.17 ± 1.66	214 ± 3.97
3DCE R-FCN [1]	60.25 ± 1.83	71.01 ± 1.19	78.99 ± 1.12	84.39 ± 0.58	87.66 ± 0.46	89.90 ± 0.70	54.62 ± 2.07	232 ± 2.65
Dense DAL 3DCE R-FCN [3]	60.61 ± 1.60	71.52 ± 0.84	79.78 ± 0.95	**85.10 ± 0.94**	88.52 ± 0.99	90.68 ± 0.82	54.41 ± 1.49	243 ± 2.30
DA-SHT Dense 3DCE (ours)	**60.97 ± 1.32**	**72.50 ± 0.81**	**79.99 ± 0.24**	84.89 ± 0.22	**88.52 ± 0.26**	**90.86 ± 0.30**	**55.15 ± 1.29**	233 ± 6.11

**Table 7:** Multi-organ lesion detection (MOLD) task results on the extracted multi-organ DeepLesion dataset. AS (%) at various FPs per image and mAP (%) were used as the evaluation metrics. std denotes the standard deviation value.

Evaluation Metric	AS@0.5 ± std	AS@1 ± std	AS@2 ± std	AS@4 ± std	AS@8 ± std	AS@16 ± std	mAP ± std
Faster R-CNN [25]	37.01 ± 0.49	47.38 ± 0.44	56.73 ± 0.89	64.69 ± 0.33	70.97 ± 0.52	75.92 ± 1.43	32.20 ± 0.42
Original R-FCN [21]	31.52 ± 0.24	41.13 ± 0.33	49.69 ± 0.86	56.38 ± 1.16	63.21 ± 1.03	67.98 ± 1.05	25.74 ± 0.48
3DCE, 9 slices [1]	36.77 ± 1.28	47.01 ± 2.39	56.80 ± 2.46	65.08 ± 2.34	70.83 ± 2.00	75.65 ± 1.82	32.43 ± 1.66
Dense DAL 3DCE R-FCN, 9 slices [3]	42.09 ± 0.80	54.80 ± 1.44	65.17 ± 0.72	71.33 ± 1.28	76.79 ± 1.13	80.39 ± 0.81	36.76 ± 1.47
Dense DKS 3DCE R-FCN, 9 slices [37]	34.00 ± 0.58	42.84 ± 1.58	53.50 ± 0.63	62.48 ± 0.09	67.90 ± 0.09	74.36 ± 1.00	29.76 ± 0.28
MP3D, 9 slices [7]	44.32 ± 1.11	53.30 ± 0.89	61.69 ± 0.60	68.16 ± 0.90	74.94 ± 0.08	80.07 ± 0.26	**43.58 ± 1.01**
DA-SHT Dense 3DCE R-FCN, 9 slices (ours)	**44.37 ± 0.12**	**58.05 ± 1.13**	**67.29 ± 0.12**	**73.88 ± 0.17**	**78.97 ± 0.14**	**82.11 ± 0.12**	40.39 ± 0.41

**Table 8:** Multi-organ lesion detection (MOLD) task results in 8 different kinds of lesions. Sensitivity (%) at 4 FPs per image ± standard deviation (std) was used as the evaluation metrics. The lesion name abbreviations can be found in Section 3.4.

	Faster R-CNN	Original R-FCN	3DCE, 9 Slices	DENSE DAL 3DCE R-FCN	Dense DKS 3DCE R-FCN	DA-SHT Dense 3DCE R-FCN (Ours)
BN	28.79 ± 1.50	16.69 ± 7.02	15.34 ± 11.99	29.52 ± 0.66	30.85 ± 1.38	40.47 ± 0.66
AB	26.68 ± 0.54	21.74 ± 1.06	26.41 ± 3.80	33.87 ± 2.66	24.84 ± 1.70	35.16 ± 2.66
ME	43.02 ± 0.12	39.22 ± 0.74	45.57 ± 7.72	50.56 ± 0.54	46.83 ± 2.90	55.26 ± 2.40
LV	34.58 ± 1.58	32.00 ± 1.98	38.79 ± 4.90	36.19 ± 2.40	30.84 ± 0.41	41.09 ± 2.28
LU	52.28 ± 0.35	52.93 ± 0.63	56.02 ± 1.89	56.23 ± 2.28	52.76 ± 1.49	58.85 ± 2.52
KD	29.13 ± 8.80	12.38 ± 1.85	25.72 ± 4.29	31.78 ± 2.52	17.01 ± 0.79	28.23 ± 6.37
ST	22.28 ± 3.50	13.58 ± 1.67	24.68 ± 1.11	26.08 ± 5.64	16.65 ± 1.34	26.56 ± 5.64
PV	20.84 ± 1.11	17.40 ± 3.54	26.94 ± 1.79	29.86 ± 0.41	18.28 ± 1.46	36.68 ± 0.41

**Table 9:** Multi-organ lesion detection (MOLD) task results in 8 different kinds of lesions. Average Precision (%) ± standard deviation (std) was used as the evaluation metric. The lesion name abbreviations can be found in Section 3.4.

	Faster R-CNN	Original R-FCN	3DCE, 9 Slices	Dense DKS 3DCE R-FCN	DENSE DAL 3DCE R-FCN	DA-SHT Dense 3DCE R-FCN (Ours)
BN	48.65 ± 4.22	37.84 ± 12.48	40.54 ± 11.42	54.05 ± 4.46	54.05 ± 4.65	59.46 ± 14.28
AB	63.5 ± 1.48	56.13 ± 2.04	64.42 ± 3.81	63.19 ± 8.84	68.40 ± 2.86	71.78 ± 2.86
ME	75.95 ± 1.42	70.99 ± 1.33	77.48 ± 1.02	79.77 ± 4.50	79.01 ± 1.43	82.44 ± 1.43
LV	69.57 ± 2.28	69.57 ± 1.60	73.91 ± 2.47	71.20 ± 1.38	79.35 ± 3.84	78.26 ± 3.84
LU	80.45 ± 2.28	79.89 ± 2.73	81.87 ± 1.68	79.32 ± 3.46	82.72 ± 2.38	84.14 ± 2.38
KD	63.08 ± 6.79	43.08 ± 5.10	58.46 ± 0.54	46.15 ± 1.83	72.31 ± 2.99	61.54 ± 2.99
ST	58.56 ± 0.78	46.85 ± 2.23	63.96 ± 1.67	51.35 ± 1.97	66.67 ± 1.33	73.87 ± 1.33
PV	57.78 ± 1.53	46.67 ± 1.22	60.00 ± 0.71	54.81 ± 0.88	68.15 ± 0.18	74.07 ± 0.18

**Table 10:** Comparison of the baseline and proposed modules during training and analysis steps. AS (%) at 4 FPs per image was used as the evaluation metric.

Method	Epoch 1	Epoch 2	Epoch 3	Epoch 4	Epoch 5	Epoch 6	Epoch 7	Epoch 8
Faster R-CNN [25]	21.40	33.90	46.98	46.34	63.39	64.13	65.34	65.08
Original R-FCN [21]	12.24	28.06	37.96	41.83	58.14	57.63	58.67	57.87
3DCE, 9 slices [1]	21.81	39.18	47.81	51.06	67.10	68.91	68.78	69.41
Dense DAL 3DCE R-FCN, 9 slices [3]	31.54	49.97	50.30	64.59	74.46	73.96	73.61	73.53
Dense DKS 3DCE R-FCN, 9 slices [37]	17.79	29.53	38.29	44.87	62.31	63.06	63.77	63.74
DA-SHT Dense 3DCE R-FCN, 9 slices (ours)	32.28	47.16	53.53	60.07	74.66	75.84	75.92	76.14

**Table 11:** Performance comparison of various methods and results from our ablation study. The “LOSS” column indicates integration with auxiliary losses, “DAL” refers to dense auxiliary losses, “SHT” represents our skipped-layer scheme, and “RANDOM” involves three arbitrary auxiliary losses from Conv1 to Conv5. Rows 5 and 6 present outcomes for the Universal Lesion Detection (ULD) task, while rows 7 through 9 detail the Multi-Organ Lesion Detection (MOLD) task.

3DCE?	DENSE?	DA?	LOSS?	Sen@2 ± std	Sen@4 ± std	AS@2 ± std	AS@4 ± std	mAP ± std
✔				78.99 ± 1.12	84.39 ± 0.58	56.80 ± 2.46	65.08 ± 2.34	32.43 ± 1.66
✔		✔		77.67 ± 0.36	83.33 ± 0.53	60.02 ± 1.02	67.32 ± 1.13	35.74 ± 1.40
✔	✔			79.36 ± 0.77	84.47 ± 0.24	63.58 ± 0.38	71.42 ± 0.26	37.99 ± 0.17
✔	✔		DAL	79.78 ± 0.95	**85.10 ± 0.94**	65.17 ± 0.72	71.33 ± 1.28	36.76 ± 1.47
✔	✔	✔	DAL	79.84 ± 0.85	84.79 ± 0.35	66.25 ± 0.33	73.20 ± 0.13	40.29 ± 0.18
✔	✔	✔	RANDOM	79.93 ± 0.87	84.69 ± 0.36	66.30 ± 0.34	73.63 ± 0.14	40.38 ± 0.40
✔	✔	✔	SHT	**79.99 ± 0.24**	84.8 ± 0.22	**67.29 ± 0.12**	**73.88 ± 0.17**	**40.39 ± 0.41**

**Table 12:** Multi-organ lesion detection (MOLD) results of DA-SHT Dense 3DCE R-FCN, 9 slices model on the extracted multi-organ DeepLesion dataset using different values of β in Equation (1). The default setting of using α = 10 is the same as that in [1,25]. AS (%) at various FPs per image and mAP (%) were used as the evaluation metrics. std denotes the standard deviation value.

α and β Value	AS@0.5 ± std	AS@1 ± std	AS@2 ± std	AS@4 ± std	AS@8 ± std	AS@16 ± std	mAP ± std
α = 10, β = 0.1	35.50 ± 0.22	47.13 ± 0.67	58.00 ± 0.04	66.69 ± 0.06	74.06 ± 0.52	77.83 ± 0.29	31.96 ± 0.15
α = 10, β = 1	**44.37 ± 0.12**	**58.05 ± 1.13**	**67.29 ± 0.12**	**73.88 ± 0.17**	**78.97 ± 0.14**	**82.11 ± 0.12**	**40.39 ± 0.41**
α = 10, β = 10	36.20 ± 0.19	48.48 ± 0.46	60.04 ± 0.73	67.04 ± 0.79	72.25 ± 0.81	77.51 ± 0.81	32.24 ± 0.37
α = 10, β = 100	37.60 ± 0.17	49.43 ± 0.52	60.44 ± 0.33	68.10 ± 0.48	75.83 ± 0.33	79.59 ± 0.20	34.42 ± 0.24

**Table 13:** Experimental results after adding different levels of Gaussian noise to the predicted depth scores generated by the depth regressor in the first stage. Multi-organ lesion detection (MOLD) task results on the extracted multi-organ DeepLesion dataset have been shown using the proposed DA-SHT Dense 3DCE R-FCN, 9 slices model. AS (%) at various FPs per image and mAP (%) were used as the evaluation metrics. std denotes the standard deviation value.

Gaussian Noise	AS@0.5 ± std	AS@1 ± std	AS@2 ± std	AS@4 ± std	AS@8 ± std	AS@16 ± std	mAP ± std
mean = 0, standard deviation = 0.01	46.24 ± 0.67	56.84 ± 0.52	67.31 ± 0.47	74.54 ± 0.16	78.78 ± 0.43	81.56 ± 0.29	40.69 ± 0.50
mean = 0, standard deviation = 0.02	44.56 ± 0.57	56.03 ± 0.27	67.37 ± 0.26	73.77 ± 0.18	78.89 ± 0.23	81.09 ± 0.09	40.39 ± 0.50
mean = 0, standard deviation = 0.1	43.81 ± 0.12	56.30 ± 0.27	66.51 ± 0.56	73.86 ± 0.75	77.77 ± 0.22	81.49 ± 0.36	39.90 ± 0.62
mean = 0, standard deviation = 0.2	43.80 ± 1.68	56.43 ± 1.00	67.24 ± 0.28	73.23 ± 0.44	78.40 ± 0.50	81.20 ± 0.59	39.53 ± 1.09

## Data Availability

The DeepLesion dataset is a publicly available dataset. The official homepage is https://nihcc.app.box.com/v/DeepLesion (accessed on 1 January 2019). The data have been anonymized and de-identified to protect patient privacy. According to the dataset’s terms of use, informed consent was obtained from all patients at the time of data collection, and the hospital has authorized the reuse of these data for research purposes. The dataset can be accessed at https://nihcc.app.box.com/v/DeepLesion (accessed on 1 January 2019).

## Supplementary Materials

The following supporting information can be downloaded at: https://www.mdpi.com/article/10.3390/bioengineering11100998/s1, Figure S1: Architecture of the 3DCE R-FCN network [1]; Figure S2: Qualitative results using the DA-SHT Dense 3DCE framework on different lesions. Predictions with scores >0.7 are shown; Figure S3: Qualitative results using the DA-SHT Dense 3DCE framework on different lesions. Predictions with scores >0.8 are shown.
