# Supplementary material for: Depth-Aware Networks for Multi-Organ Lesion Detection in Chest CT Scans
Source: Bioengineering (Basel). 2024 Oct 3;11(10):998. doi: 10.3390/bioengineering11100998 (PMC11503988; doi:10.3390/bioengineering11100998)
Supplement: Supplementary file 1 [file bioengineering-11-00998-s001.zip › bioengineering-3095014-supplementary.pdf]

Depth-aware Networks for Multi-organ Lesion Detection in Chest CT scans

Han Zhang and Albert C. S. Chung

June 2024

1 Supplementary Materials

References

[1] K. Yan, M. Bagheri, and R. M. Summers, "3d context enhanced region-based convolutional neural network for end-to-end lesion detection," in *International Conference on Medical Image Computing and Computer-Assisted Intervention*. Springer, 2018, pp. 511–519.

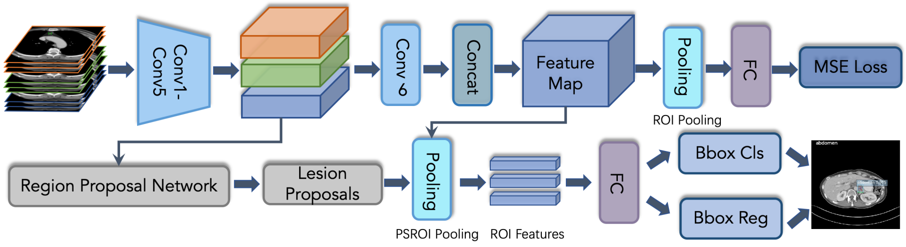

Fig. S1: Architecture of the 3DCE R-FCN network [1].

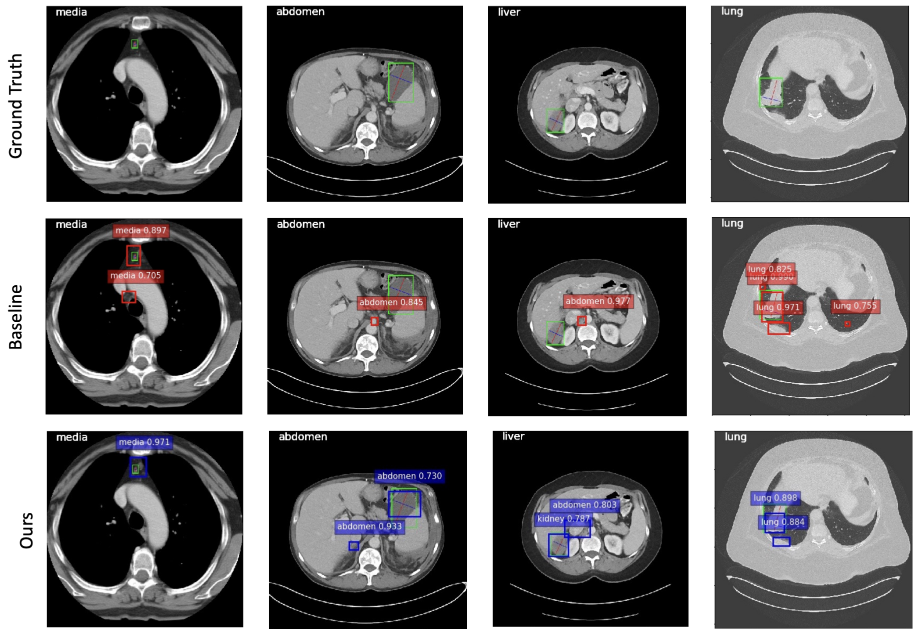

Fig. S2: Qualitative results using the DA-SHT Dense 3DCE framework on different lesions. The bounding boxes in red and blue highlight the categorical and confidence scores of the automatic detection results, respectively. Predictions with scores > 0.7 are shown.

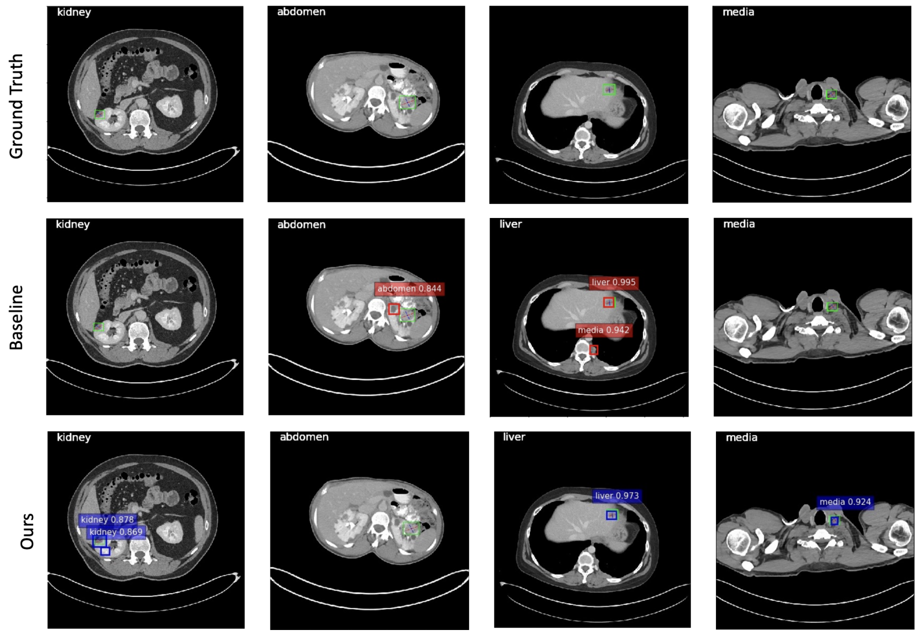

Fig. S3: Qualitative results using the DA-SHT Dense 3DCE framework on different lesions. The bounding boxes in red and blue highlight the categorical and confidence scores of the automatic detection results, respectively. Predictions with scores > 0.8 are shown.
